# Antagonizing Arachidonic Acid-Derived Eicosanoids Reduces Inflammatory Th17 and Th1 Cell-Mediated Inflammation and Colitis Severity

**DOI:** 10.1155/2014/917149

**Published:** 2014-07-17

**Authors:** Jennifer M. Monk, Harmony F. Turk, Yang-Yi Fan, Evelyn Callaway, Brad Weeks, Peiying Yang, David N. McMurray, Robert S. Chapkin

**Affiliations:** ^1^Program in Integrative Nutrition & Complex Diseases, Center for Translational Environmental Health Research, Texas A&M University, College Station, TX, USA; ^2^Department of Nutrition & Food Science, Texas A&M University, College Station, TX, USA; ^3^Department of Veterinary Pathobiology, Texas A&M University, College Station, TX, USA; ^4^University of Texas MD Anderson Cancer Center, Houston, TX 77030, USA; ^5^Department of Microbial Pathogenesis and Immunology, Texas A&M University System Health Science Center, College Station, TX, USA

## Abstract

During colitis, activation of two inflammatory T cell subsets, Th17 and Th1 cells, promotes ongoing intestinal inflammatory responses. n-6 polyunsaturated fatty acid- (PUFA-) derived eicosanoids, such as prostaglandin E_2_ (PGE_2_), promote Th17 cell-mediated inflammation, while n-3 PUFA antagonize both Th17 and Th1 cells and suppress PGE_2_ levels. We utilized two genetic mouse models, which differentially antagonize PGE_2_ levels, to examine the effect on Th17 cells and disease outcomes in trinitrobenzene sulfonic acid- (TNBS-) induced colitis. *Fat-1* mice contain the *ω*3 desaturase gene from *C. elegans* and synthesize n-3 PUFA *de novo*, thereby reducing the biosynthesis of n-6 PUFA-derived eicosanoids. In contrast, *Fads1* Null mice contain a disrupted Δ5 desaturase gene and produce lower levels of n-6 PUFA-derived eicosanoids. Compared to Wt littermates, *Fat-1* and *Fads1* Null mice exhibited a similar colitic phenotype characterized by reduced colonic mucosal inflammatory eicosanoid levels and mRNA expression of Th17 cell markers (IL-17A, ROR*γτ*, and IL-23), decreased percentages of Th17 cells and, improved colon injury scores (*P* ≤ 0.05). Thus, during colitis, similar outcomes were obtained in two genetically distinct models, both of which antagonize PGE_2_ levels via different mechanisms. Our data highlight the critical impact of n-6 PUFA-derived eicosanoids in the promotion of Th17 cell-mediated colonic inflammation.

## 1. Introduction

Inflammatory bowel disease (IBD) manifests as two clinical conditions, ulcerative colitis (UC) and Crohn's disease (CD). The induction and persistence of chronic inflammation during IBD is attributed to the activation of two inflammatory T cell subsets (Th17 and Th1 cells) and production of their signature cytokines, IL-17 and IFN*γ*, respectively [1–4]. The role of Th17 cells in the pathogenesis of IBD has been documented in humans with active disease [5–7] and in mouse models in which disease severity was reduced by blockade or deficiency of IL-23 and/or IL-17 [8, 9].

A recent case-control study found that high intakes of n-6 polyunsaturated fatty acids (PUFA) increased the risk of developing UC [10] which was attributed, in part, to the immunomodulatory effects of arachidonic acid- (AA-) derived eicosanoids [11]. In IBD patients (CD and UC) increased levels of AA and its eicosanoid metabolites, such as PGE_2_, leukotriene B_4_ (LTB_4_), and thromboxane B_2_ (TXB_2_), were found in the mucosa of the gastrointestinal tract [12–17]. In UC patients, the concentration of PGE_2_ increased in proportion to the degree of mucosal injury or disease severity [17]. Moreover, in IBD patients, the combination of PGE_2_, IL-23, and IL-1*β* works synergistically to enhance IL-17A secretion from CD161^+^ CD4^+^ T cells [18] which infiltrate the gastrointestinal tract [19–21]. In the trinitrobenzene sulfonic acid- (TNBS-) induced mouse colitis model, which induces T cell-mediated immune responses within the colonic mucosa [22] and is driven by inflammatory Th17 cells [23], both serum and colonic mucosal PGE_2_ levels were elevated [24]. PGE_2_ was shown to exacerbate colonic inflammatory processes and colitis severity in this model through the activation of the IL-23/IL17 axis and by increasing local Th17 cell numbers [25]. Through alterations in the cytokine microenvironment, PGE_2_ can influence inflammatory T cell development directly by skewing naïve T cell differentiation and effector function toward the production of proinflammatory Th17 and Th1 cell subsets [18, 26–29] and indirectly by inducing antigen presenting cells to favor IL-23 production [25, 30, 31], thereby promoting the differentiation and maintenance of Th17 cells. Other n-6 PUFA-derived eicosanoids have also been shown to promote Th17 cell development [32], thereby demonstrating partial functional redundancy in the immunomodulatory effects of the AA-derived eicosanoid profile. Collectively, these data indicate that AA-derived eicosanoids may drive the activation of Th17 cells during IBD and any treatment strategy designed to antagonize their mucosal levels could reduce Th17 cell activation and the severity of the disease phenotype.

Fish oil (FO) derived long chain n-3 PUFA exert anti-inflammatory effects [33–35] and have been shown to enhance remission of chronic intestinal inflammation [36]. Moreover, an estimated 50% of IBD patients utilize self-prescribed oral complementary alternative medicines/diets, such as FO [37]. Dietary n-3 PUFA accumulate in cell membranes, partly at the expense of AA, thereby reducing the available substrate for the synthesis of AA-derived eicosanoids [38–41] while concomitantly serving as substrates for the production of n-3 PUFA-derived anti-inflammatory resolvins, docosatrienes, and neuroprotectins [42]. Further, n-3 PUFA have been demonstrated to reduce splenic CD4^+^ T cell* ex vivo* polarization into Th1 [43, 44] and Th17 cells [45]. Therefore, n-3 PUFA may suppress colitis-associated Th17 cell activation, in part, by reducing mucosal AA-derived eicosanoid levels. To test this hypothesis, we utilized two genetic mouse models which antagonize AA-derived eicosanoid production: (i) the* Fat-1* transgenic mouse which produces long chain n-3 PUFA* de novo* [46] and exhibits reduced colonic AA-derived eicosanoid levels [47] and (ii) the* Fads1* Null mouse, which exhibits systemic disruption of the* Fads1* (Δ5 desaturase) gene, reciprocally altering the tissue level of dihomo-*γ*-linolenic (DGLA) and AA, resulting in decreased AA-derived eicosanoid levels [48]. In this study, we determined the effect of antagonizing AA-derived eicosanoids on local [colon and mesenteric lymph node (MLN)] and systemic (splenic) levels of the relevant CD4^+^ T cell effector subsets (Th17, Th1, Th2, and Tregs) and Th17 cell-mediated colonic cytokine expression in response to TNBS-induced colitis.

## 2. Materials and Methods 

### 2.1. Animals and Diets

*Fads1* and* Fat-1* transgenic mice, both on a C57BL/6 background, were generated in collaboration with the Texas Institute for Genomic Medicine (Texas A&M University) and Dr. Jing Kang (Harvard University), respectively.* Fads1* knockout mice [genotypes: wild-type (Wt), heterozygous (Het), and null (Null)] represent a Δ5 desaturase knockout strain that produces AA deficiency without the underlying complication of essential fatty acid deficiency [i.e., linoleic acid (LA) or DGLA] [48].* Fat-1* transgenic mice (genotypes: Wt and* Fat-1*) synthesize long chain n-3 PUFA* de novo* [46]. Littermate specific pathogen-free male and female mice from both strains were genotyped, phenotyped, and housed as previously described [46–48]. All mice were fed a commercial 10% safflower oil diet (D03092902R; Research Diets, New Brunswick, NJ, USA), wherein GC fatty acid analysis of the diet confirmed that it is free of AA and contained trace levels of n-3 PUFA (0.17% *α*-linolenic acid) but was adequate in all other nutrients. All procedures adhered to U.S. Public Health Service Policy and were approved by the Institutional Animal Care and Use Committee at Texas A&M University.

### 2.2. Colitis Induction and Histological Scoring

Colonic inflammation was induced by exposure to 2,4,6-trinitrobenzene sulfonic acid (TNBS; Sigma Aldrich, St. Louis, MO, USA) as previously described [49]. In brief, mice were individually housed and 100 *μ*L of a 1% (w/v) presensitizing dosage of TNBS dissolved in a 4 : 1 volume ratio of acetone and olive oil (Azienda, Florence, Italy) was applied topically onto a shaved 1.5 × 1.5 cm field between the shoulders. The site was selected to prevent the animals from ingesting the TNBS which could induce oral tolerance [49]. After 7 d, mice were anesthetized with isoflurane to effect and were under anesthesia for ≤5 min during which time a 100 *μ*L enema containing 2.5% (w/v) TNBS in a 1 : 1 volume ratio of water and absolute ethanol was administered. Saline control mice were exposed to the presensitization vehicle topically (4 : 1 volume ratio of acetone and olive oil) followed 7 d later by a 100 *μ*L saline enema. All mice were sacrificed 3 d after TNBS enema by CO_2_ asphyxiation. Colons were excised (distal to the cecum and proximal to the anus) and flushed with sterile PBS and the mucosa was scraped from one longitudinal colon half for gene expression analysis. The other longitudinal colon half was fixed in 4% paraformaldehyde, Swiss-rolled, paraffin embedded, and stained with hematoxylin and eosin. The degree of colon injury (score 0–3 per colon region, i.e, proximal, middle, and distal) was graded in a blinded manner by a board-certified pathologist (B. Weeks) in accordance with the criteria outlined previously [47].

### 2.3. Flow Cytometry Analysis of T Cell Subsets

Splenic and MLN mononuclear cells were isolated by lympholyte-M enrichment (Cedarlane, Burlington, NC, USA) as described [50]. Colonic tissues were digested using Type IV collagenase (Sigma Aldrich) as described elsewhere [51] and lymphocytes were enriched over 70/45% Percoll gradient (Sigma Aldrich). Surface and intracellular staining were performed as reported previously [45, 52]. Cells were surface-stained with APC-anti-CD4 (clone L3T4, eBioscience, San Diego, CA, USA) followed by intracellular detection of PE-anti-FOXP3 (clone FJK-16s), PE-anti-IL-17A (clone eBio17B7), PE-anti-IFN*γ* (clone XMG1.2), or PE-anti-IL-4 (clone 11B11) (eBioscience). Isotype controls utilized were PE-IgG2_a_ *κ*, PE-IgG1*κ*, and APC-IgG2_b_ *κ* (eBioscience). Flow cytometric analysis was conducted using a BD Accuri C6 flow cytometer (BD Bioscience, San Jose, CA, USA).

### 2.4. RNA Isolation and Measurement of mRNA Expression

RNA was isolated from colon mucosal scrapings using the RNA 4-PCR kit (Ambion/Life Technologies, Grand Island, NY, USA). Real-time RT-PCR was used to quantify mRNA expression and amplification was performed using the Taqman Universal PCR master mix and Taqman gene expression kits (Applied Biosystems/Life Technologies, Grand Island, NY, USA) were used for amplification of IL-1*β* (Mm00434228_m1), IL-6 (Mm00446190_m1), IL-17A (Mm00439618_m1), IL-17F (Mm00521423_m1), IL-21 (Mm00517640_m1), IL-22 (Mm01226722_g1), IL-23 (Mm00518984_m1), IL23R (Mm_00519943_m1), IFN*γ* (Mm01168134_m1), IL-27 (Mm00461162_m1), TNF*α* (Mm00443260_g1), CCL2 (MCP-1, Mm00441242_m1), IL-4 (Mm00445259_m1), IL-10 (Mm00439614_m1), TGF*β*1 (Mm01178820_m1), Rorc (ROR*γ* *τ*, Mm01261022_m1), Tbx21 (T-bet, Mm00450960_m1), FOXP3 (Mm00475162_m1), CCL20 (Mm01268754_m1), and CCR6 (Mm99999114_s1). Amplification of mRNA (fluorescence) was recorded over 40 cycles and the corresponding cycle numbers (Ct) were used to calculate mRNA expression according to the calculation: 2^(40−Ct)^. Target gene expression was normalized to ribosomal 18S expression (Mm03928990_g1).

### 2.5. Colonic Mucosal Eicosanoid Profiling

Eicosanoids were extracted from colonic scraped mucosa from TNBS-treated* Fat-1* and* Fads1* mice as previously described [47, 48, 53]. Liquid chromatography/tandem mass spectroscopic analyses were performed using a QuattroUltima mass spectrometer (Waters, Milford, MA, USA) equipped with an Agilent 1100 binary pump high-performance liquid chromatography system (Agilent Technologies, Santa Clara, CA, USA) according to a modified version of the method of Yang et al. [53]. Eicosanoids of interest were chromatographically separated using a Luna 3 *μ*m phenyl-hexyl 4.6 × 100 mm analytic column (Phenomenex, Torrance, CA, USA) [48]. Eicosanoids were detected and quantified by multiple reaction mode monitoring of the transitions *m*/*z* as described elsewhere [48, 54].

### 2.6. Statistics

The predetermined upper limit of probability for statistical significance throughout this investigation was *P* ≤ 0.05, and analyses were conducted using the SAS system for Windows Version 9.0 (SAS Institute, Cary, NC, USA). Data were analyzed by either one-way ANOVA or two-way ANOVA (main effects: genotype and treatment) followed, if justified, by testing using Least Squares Means. Data sets not exhibiting a normal distribution were analyzed using the Kruskal-Wallis test (*χ* ^2^ approximation) followed, if justified, by the statistical probability outcome (*P* ≤ 0.05) using Wilcoxon two-sample testing.

## 3. Results

### 3.1. Colonic Mucosal Inflammatory AA-Derived Eicosanoid Profile Is Antagonized in Fat-1 and Fads1 Null Mice

The colonic mucosal eicosanoid profiles from TNBS-treated* Fat-1* (Table 1) and* Fads1* (Table 2) mice are shown. In* Fat-1* mice, colonic mucosal levels of n-6 PUFA-derived eicosanoids, specifically PGE_2_, prostaglandins (PG) D_2_ and F_2*α*_, 15-hydroxy-eicosatetraenoic acid (HETE), arachidonoyl ethanolamine (AEA), and arachidonoyl glycerol (AG), were reduced significantly, whereas the n-3 PUFA-derived PGE_3_ was increased compared to Wt (*P* < 0.05). In* Fads1 *Null mice, increased levels of PGE_1_ and decreased levels PGE_2_, D_2_, F_*α*_, thromboxane (TX) B_2_, 5-HETE, AEA, and 2AG (*P* < 0.05) were observed compared to Wt littermates. Collectively, these data demonstrate that the local inflammatory (n-6 PUFA-derived) eicosanoid profile in response to TNBS-induced colitis is antagonized in both genetic mouse models via two different mechanisms, that is, by increasing n-3 PUFA content in* Fat-1* mice and by inducing AA deficiency in* Fads1* Null mice, as seen previously [47, 48].

### 3.2. Fat-1 Mice Are More Resistant to TNBS-Induced Colon Injury

**Figure 1:**
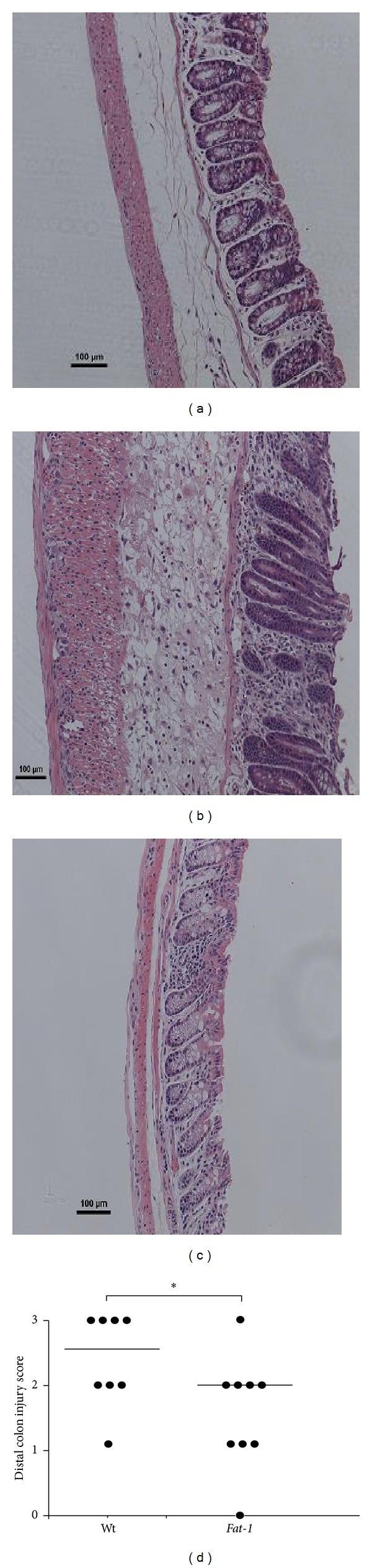
The degree of distal colon histological injury/damage in* Fat-1* and Wt TNBS-treated mice (*n* = 8-9/genotype). ((a) and (b)) Representative images (100x magnification, scale bar = 100 *μ*m) of TNBS-treated* Fat-1* and Wt mice, (c) Wt saline control treated (injury score = 0) distal colons, and (d) injury scores (0–3) in the distal colon from Wt and* Fat-1* TNBS-treated mice, respectively. Data were analyzed using the Kruskal-Wallis test (*P* ≤ 0.05). Median values are shown and significant differences between genotypes are marked with an asterisk (*P* ≤ 0.05).

Body weight and colon length at the time of sacrifice are shown in Supplemental Table  1 (see Supplementary Material available online at http://dx.doi.org/10.1155/2014/917149). Compared to saline controls, TNBS-treated mice exhibited lower body weights and colon shortening, but they did not differ between genotypes (*P* > 0.05). The degree of colon injury following exposure to TNBS was assessed based on colonic histological changes in a blinded manner by a board-certified pathologist (B. Weeks). Representative distal colon images from Wt and* Fat-1* saline control and TNBS-treated mice are shown (Figures 1(a)–1(c)). The saline control treatment did not induce colonic damage and both Wt and* Fat-1* mice exhibited an injury score of 0 in all regions of the colon. In TNBS-treated mice, injury scores were lower in the proximal and middle regions of the colon compared to the distal colon; however, they did not differ between Wt and* Fat-1* mice (proximal colon, *P* = 0.45, and middle colon, *P* = 0.54, results not shown). However, within the distal colon (site of TNBS administration via rectal enema), the degree of colonic injury was significantly reduced in* Fat-1* mice compared to Wt (*P* = 0.05, Figure 1(d)). This outcome is consistent with the TNBS model exerting the majority of histopathological damage within the distal colon [55] and with the ability of n-3 PUFA to enhance the resolution of inflammatory processes and reduce colonic injury by promoting mucosal repair [47, 56].

### 3.3. Colitis-Associated Changes in CD4^+^ T Cell Subsets in Local and Systemic Anatomical Sites in Wt and Fat-1 Mice

**Figure 2:**
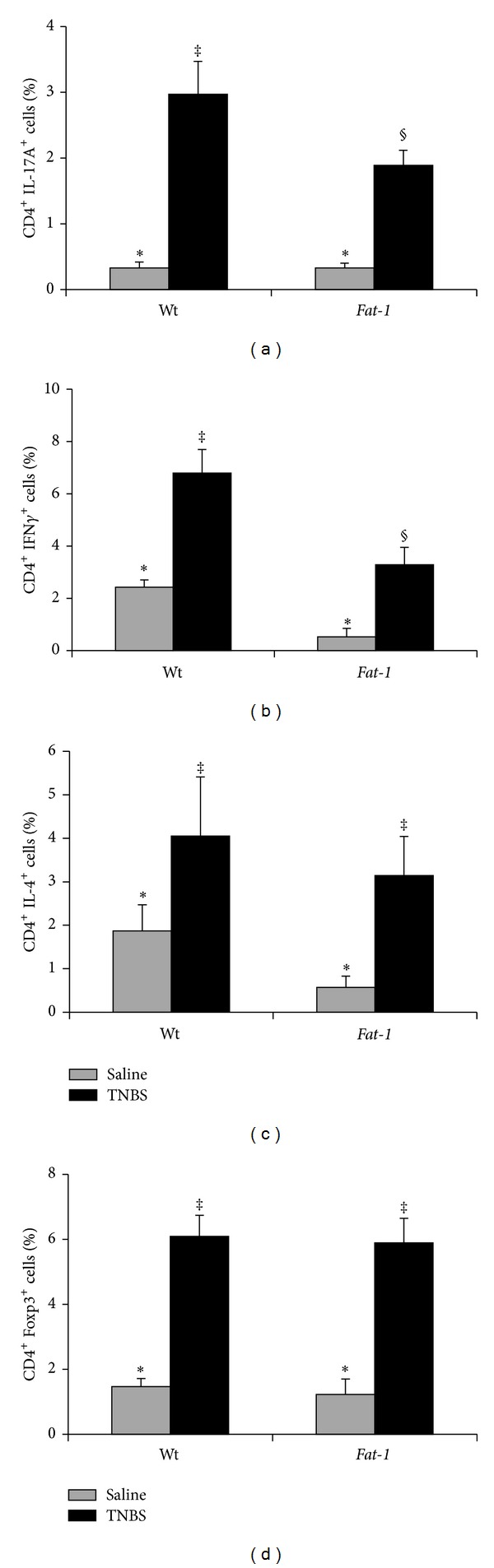
Percentage of colon lamina propria CD4^+^ T cell subsets in Wt and* Fat-1* TNBS-treated mice (black bars, *n* = 8–16 pooled samples/genotype comprised of 2-3 colons) and saline controls (grey bars, *n* = 4 pooled samples/genotype). (a) Th17 cells (CD4^+^ IL-17A^+^), (b) Th1 cells (CD4^+^ IFN*γ* ^+^), (c) Th2 cells (CD4^+^ IL-4^+^), and (d) Tregs (CD4^+^ Foxp3^+^). Data were analyzed by two-way ANOVA (main effects: genotype and treatment); bars represent means ± SEM. Bars not sharing a symbol differ (*P* ≤ 0.05).

**Figure 3:**
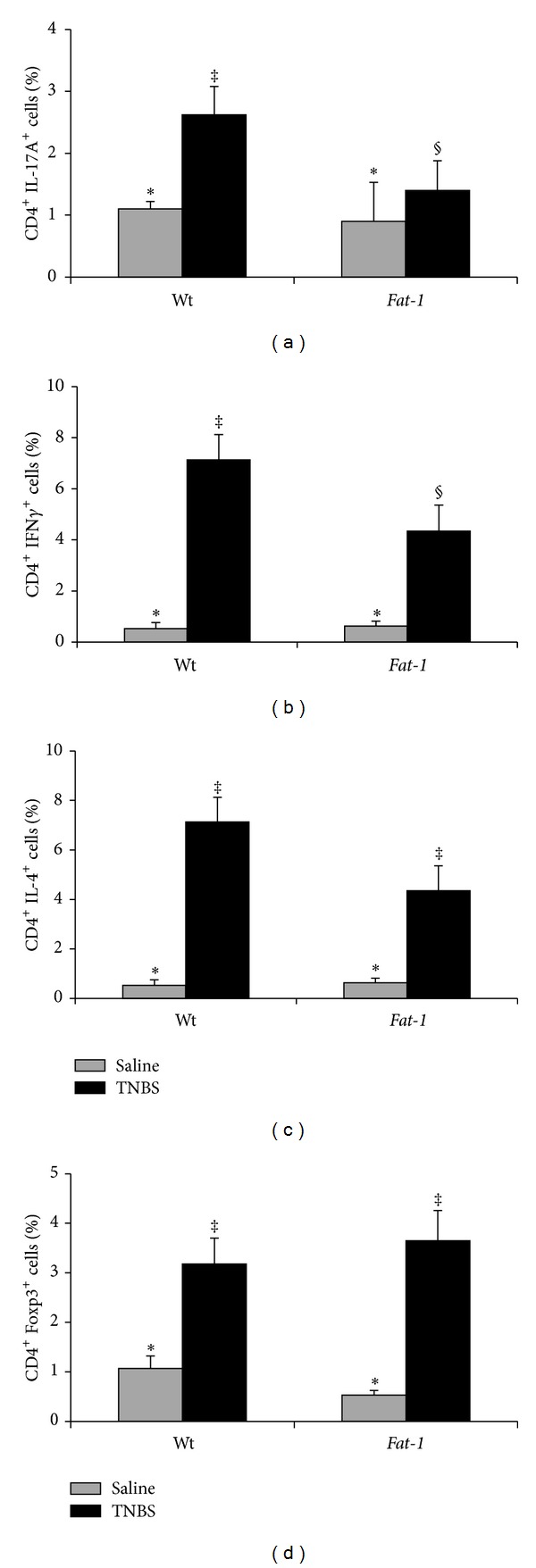
Percentage of mesenteric lymph node (MLN) CD4^+^ T cell subsets in Wt and* Fat-1* TNBS-treated mice (black bars, *n* = 9–12 pooled samples/genotype comprised of 2–4 MLNs) and saline controls (grey bars, *n* = 4 pooled samples/genotype). (a) Th17 cells (CD4^+^ IL-17A^+^), (b) Th1 cells (CD4^+^ IFN*γ* ^+^), (c) Th2 cells (CD4^+^ IL-4^+^), and (d) Tregs (CD4^+^ Foxp3^+^). Data were analyzed by two-way ANOVA (main effects: genotype and treatment); bars represent means ± SEM. Bars not sharing a symbol differ (*P* ≤ 0.05).

**Figure 4:**
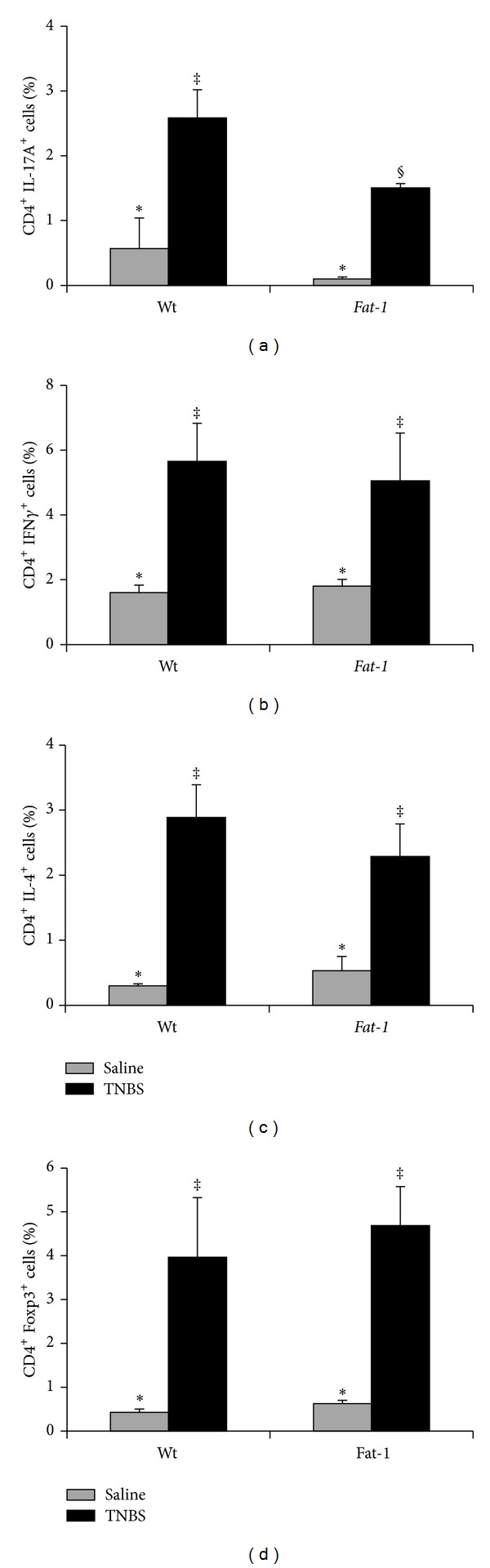
Percentage of splenic CD4^+^T cell subsets in Wt and* Fat-1* TNBS-treated mice (black bars, *n* = 10–12 spleens/genotype) and saline controls (grey bars, *n* = 4/genotype). (a) Th17 cells (CD4^+^ IL-17A^+^), (b) Th1 cells (CD4^+^ IFN*γ* ^+^), (c) Th2 cells (CD4^+^ IL-4^+^), and (d) Tregs (CD4^+^ Foxp3^+^). Data were analyzed by two-way ANOVA (main effects: genotype and treatment). Bars represent means ± SEM. Bars not sharing a symbol differ (*P* ≤ 0.05).

Following the induction of colitis, the effect of n-3 PUFA on the resident CD4^+^ T cell effector subset populations (i.e., Th1, Th2, Th17, and Treg) was documented both locally (colon lamina propria and MLN) and systemically (spleen). Representative dot plots for T cell subsets isolated from Wt TNBS-treated colon, MLN, and spleen are shown in Supplemental Figure  1. In all tissue sites, TNBS exposure increased the percentage of all CD4^+^ T cell subsets compared to the saline control group (treatment: *P* < 0.05, Figures 2–4). Within the colon, the percentages of proinflammatory Th17 and Th1 cells were reduced in* Fat-1* mice compared to Wt (*P* < 0.05, Figures 2(a) and 2(b)), whereas the percentages of Th2 and Treg cells did not differ between groups (*P* > 0.05, Figures 2(c) and 2(d)). In the MLN, which drains and is anatomically proximal to the inflamed colon, the percentages of both Th17 and Th1 cells were reduced in* Fat-1* mice compared to Wt (*P* < 0.05) (Figures 3(a) and 3(b)), whereas Treg and Th2 cells were unaffected (*P* > 0.05, Figures 3(c) and 3(d)). In the spleen, only the percentage of Th17 cells was reduced in the* Fat-1* mouse (*P* < 0.05) compared to Wt (Figure 4(a)). Splenic Th1, Th2, and Treg cell populations did not differ between groups (*P* > 0.05, Figures 4(b)–4(d)). Collectively, these data demonstrate that proinflammatory Th17 and Th1 cells are selectively antagonized by n-3 PUFA in both local and systemic sites during colitis.

### 3.4. The Colonic Mucosal Cytokine Microenvironment Is Modified in a Manner Consistent with Reduced Th17 Cell Activation in Fat-1 Mice

To gain insight into how effector T cell populations are changing in response to TNBS-induced colitis, gene expression of critical transcription factors and cytokines that make up the inflammatory colonic milieu were assessed in both Wt and* Fat-1* TNBS-treated mice (Table 3). Colonic mRNA expression of ROR*γ* *τ*, the master transcription factor that directs both the differentiation of Th17 cells and the expression of hallmark Th17 cytokines [57], was reduced significantly in* Fat-1* mice (*P* = 0.03). Conversely, n-3 PUFA had no significant effect on the mRNA expression of other key transcription factors associated with other T cell subsets, namely, Foxp3 (Tregs) and T-bet (Th1 cells) (*P* > 0.05). Gene expression of the Th17 cell signature cytokine, IL-17A, was decreased significantly in the* Fat-1* mouse versus Wt (*P* = 0.04), whereas IL-17F and IL-22 did not differ between groups (*P* > 0.05). Additionally, mRNA expression of IL-21, which promotes Th17 cell differentiation and proliferation [58, 59] and controls both Th1 and Th17 cell responses [2, 60–62], was reduced in the* Fat-1* mouse (*P* = 0.01), consistent with the reduced percentage of Th1 and Th17 cells in the colon lamina propria (Figures 2(b) and 2(c)). Colonic mRNA expression of IL-23 showed a trend towards a significant reduction in the* Fat-1* mouse (*P* = 0.06), whereas the expression of IL-23R did not differ between groups (*P* = 0.95). Interestingly, n-3 PUFA significantly upregulated mRNA expression of IL-27 (*P* = 0.04), a key cytokine that has been shown to antagonize Th17 cell development [60, 62]. Consistent with the anti-inflammatory effects of n-3 PUFA [33–35], colonic mRNA expression of IL-10 was upregulated in the* Fat-1* mouse (*P* = 0.04), whereas TGF*β*1 expression was unaffected (*P* = 0.44).* Fat-1* mRNA levels of classic inflammatory cytokines (IL-1*β* and TNF*α*) and chemokines (MCP-1) were reduced compared to Wt levels (*P* < 0.05), whereas colonic mRNA levels of IL-6, IFN*γ*, and IL-4 did not differ between genotypes (*P* > 0.05).

### 3.5. TNBS Colitis Induced Colon Injury Is Reduced in Fads1 Null Mice

**Figure 5:**
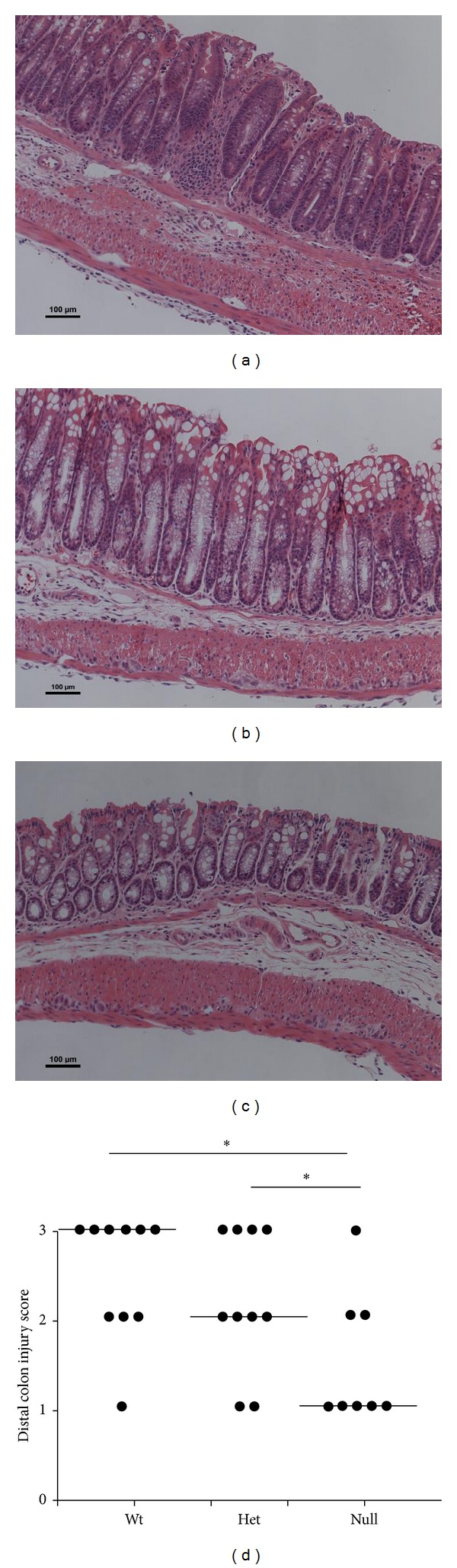
Distal colon histological injury/damage scores assessed in Wt, Het, and Null* Fads1 *TNBS-treated mice (*n* = 8–10/genotype) in a blinded manner by a board-certified pathologist (B. Weeks). ((a)–(c)) Representative images (100x magnification, scale bar = 100 *μ*m) of Wt, Het, and Null* Fads1 *TNBS-treated distal colons, respectively. (d) Distal colon injury scores (0–3). Data were analyzed using the Kruskal-Wallis test (*P* ≤ 0.05) followed by Wilcoxon two-sample testing. Median values are shown and significant differences between genotypes are marked by an asterisk (*P* ≤ 0.05).

The degree of TNBS-induced colon injury was assessed histologically in* Fads1* Wt, Het, and Null mice (representative distal colon images, Figures 5(a)–5(c)). Histological injury scores were 0 throughout the colon in saline control treated mice from all three* Fads1* genotypes. Within TNBS-treated mice, the degree of colon injury did not differ between genotypes in the proximal (*P* = 0.10) and middle (*P* = 0.31) regions of the colon. However, distal colon injury scores were reduced significantly in* Fads1* Null mice compared to both Wt and Het mice (*P* = 0.04) (Figure 5(d)). Colon length and final body weights were lower in TNBS-treated mice compared to saline controls but did not differ between genotypes (*P* > 0.05) as shown in Supplemental Table  2. These data suggest that antagonizing colon mucosal eicosanoid levels via AA deficiency reduces the severity of colon histological structural damage induced during TNBS colitis.

### 3.6. Changes in Splenic T Cell Subsets in the Fads1 Mouse

**Figure 6:**
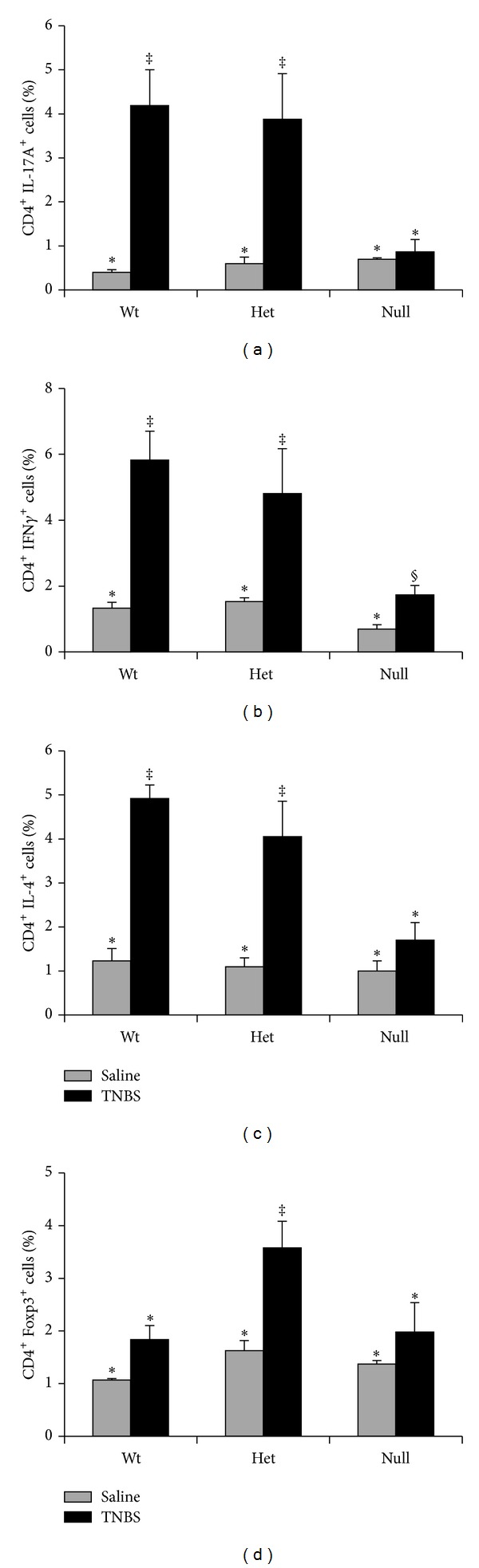
Percentage of splenic CD4^+^ T cell subsets in Wt, Het, and Null* Fads1* TNBS-treated mice (black bars, *n* = 6–9 mice/genotype) and saline controls (grey bars, *n* = 3-4 mice/genotype). (a) Th17 cells (CD4^+^ IL-17A^+^), (b) Th1 cells (CD4^+^ IFN*γ* ^+^), (c) Th2 cells (CD4^+^ IL-4^+^), and (d) Tregs (CD4^+^ Foxp3^+^). Data were analyzed by two-way ANOVA (main effects: genotype and treatment). Bars represent means ± SEM. Bars not sharing a symbol differ (*P* ≤ 0.05).

Due to limitations in mouse numbers and the necessity to pool colons to obtain sufficient cells for analysis, quantification of T cell subsets in the colon and MLN was not possible. Therefore, changes in systemic T cell subsets (i.e., Th1, Th2, Th17, and Treg) following exposure to TNBS were determined in the spleen and representative dot plots are shown in Supplemental Figure  2. As expected, TNBS treatment increased all splenic CD4^+^ T cell subsets compared to saline controls (*P* < 0.05). The percentages of splenic Th17 and Th1 cells were reduced in* Fads1* Nullmice compared to both Wt and Het mice (*P* < 0.05, Figures 6(a) and 6(b)). Interestingly, the percentage of splenic Th2 cells was also reduced in the* Fads1* Null mouse compared to both Wt and Het TNBS-treated mice (*P* = 0.04, Figure 6(c)), whereas splenic Tregs were unaffected (Wt versus Null, *P* > 0.05, Figure 6(d)). There was no difference between Wt and Het mice in the percentage of splenic Th17, Th1, and Th2 cells (Figures 6(a)–6(c)), whereas the percentage of splenic Treg cells was increased in Het mice compared to both Wt and* Fads1 *Null (*P* = 0.008, Figure 6(d)).

### 3.7. The Colonic Mucosal Cytokine Microenvironment Is Modified in a Manner Consistent with Reduced Th17 Cell Activation in Fads1Null Mice

The mRNA expression levels of key cytokines and transcription factors related to specific T cell subsets and inflammatory status in the colons of TNBS-treated* Fads1* mice are shown in Table 4. mRNA levels did not differ between Wt and Het mice for any genes except the Th1 cell master transcription factor Tbet, which was reduced in both Het and Null mice compared to Wt (*P* = 0.004). Additionally, mRNA expression of the Th17 cell master transcription factor ROR*γ* *τ* was reduced in* Fads1 *Null mice compared to Wt (*P* = 0.03). Further, in* Fads1* Null mice, mRNA levels of the Th17 cell signature cytokines, IL-17A and IL-17F, and IL-23, which maintains an established Th17 cell phenotype, were all reduced compared to Wt (*P* < 0.05). mRNA levels of the inflammatory mediator IL-6 were also reduced in* Fads1 *Null mice compared to Wt (*P* < 0.05). Collectively, these data indicate that, in mice devoid of AA-derived eicosanoids, colonic mRNA levels of Th17 cell related cytokines and transcription factors are suppressed following TNBS treatment, coinciding with an improved clinical outcome in* Fads1* Null mice.

## 4. Discussion

In the TNBS colitis model, PGE_2_ has been shown to exacerbate colonic inflammatory processes and colitis severity through activation of the IL-23/IL17 axis and by increasing local Th17 cell numbers [18, 25, 30, 31]. Moreover, PGE_2_ coordinates locally with cytokines present within the tissue microenvironment to directly promote Th17 cell differentiation and effector function [18, 26–29]. Further, other n-6 PUFA-derived eicosanoids have also been shown to promote Th17 cell development [32]. In* Fat-1* mice (Table 1), levels of n-6-derived eicosanoids were reduced and n-3 PUFA-derived PGE_3_ levels were increased compared to Wt littermates. Additionally, in* Fads1* Null mice, the biosynthesis of AA-derived eicosanoids was reduced (Table 2) and colonic mucosal PGE_2_ levels were negligible compared to Wt. Interestingly, despite the different mechanisms through which colonic tissue AA-derived eicosanoid levels, including PGE_2_, were antagonized in the two genetic mouse models utilized in this study, the TNBS-induced inflammatory phenotype observed in both models was similar. This highlights the critical role which PGE_2_ and other AA-derived eicosanoids may be playing in activating inflammatory T cell subsets and perpetuating colitis-associated inflammation and disease severity. In both mouse models (*Fat-1 *transgenic and* Fads1* Null mice), colonic mucosal AA-derived eicosanoid levels were reduced (Tables 1 and 2), colon injury scores improved (Figures 1(d) and 5(d)), the percentage of splenic inflammatory Th17 cells was decreased (Figures 4(a) and 6(a)), and colonic mucosal mRNA expression of Th17 cell markers and inflammatory mediators (IL-17A, ROR*γ* *τ*, IL-23, and MCP-1) were reduced (Tables 3 and 4). Further, in both models, loss of AA-derived eicosanoids was accompanied by reduced Th17 cell numbers and a remodeled colonic mucosal gene expression profile that was consistent with suppressed inflammatory potential and Th17 cell polarization, activation, and maintenance [2, 9, 57, 58, 61–65]. These changes coincided with reduced TNBS-induced mucosal injury, thereby demonstrating the critical roles which AA and its metabolites, for example, PGE_2_, and Th17 cells play in perpetuating TNBS colitis severity. The similarities between the two models support the conclusion that the combination of inflammatory cytokines and noncytokine immunomodulators (i.e., AA-derived eicosanoids) present in the local inflammatory microenvironment during T cell differentiation and activation determines the ultimate phenotype of Th17 cells, as previously demonstrated in the TNBS model [26].

Enriched n-3 PUFA tissue levels in* Fat-1* mice [46] corresponded to reduced colitis severity and the decreased percentages of proinflammatory Th17 and Th1 cells within the colon lamina propria (Figures 2(a) and 2(b)), the MLN (Figures 3(a) and 3(b)), and the spleen (Figures 4(a) and 4(b)). These results demonstrate that the activation and/or polarization of T cell subsets known to drive IBD [1–4] are directly antagonized by n-3 PUFA both locally and systemically. Conversely, the percentages of Tregs and Th2 cells were unaffected (Figures 2–4), indicating that the beneficial effect of n-3 PUFA was likely not due to enhanced Treg function or a shift in polarization from Th1 to Th2 cells. Interestingly, in* Fat-1* mice, changes in the colonic gene expression profile were consistent with reduced Th17 cell activation, proliferation, maintenance, and inflammatory capacity [57, 63, 65] evidenced by reduced expression of IL-17A, IL-21, IL-23, MCP-1, IL-1*β*, and TNF*α* (Table 3) and increased expression of the Th17 cell-antagonizing cytokine, IL-27, and the anti-inflammatory cytokine, IL-10.

In* Fads1* Null mice, due to the limitations in cell numbers, only splenic T cell subsets, representative of the systemic response, were assessed and the percentages of Th17, Th1, and Th2 cells were reduced compared to Wt littermates (Figures 6(a)–6(d)). Interestingly, the percentage of splenic Tregs was not affected in* Fads1 *Null mice compared to Wt and was actually elevated in Het mice (Figure 6(d)), indicating that not all CD4^+^ T cell effector subsets are reduced in* Fads1* Null mice, as regulatory capabilities appear to be sustained. Moreover, the percentage of all T cell subsets detected in* Fads1* Null saline control treated mice did not differ from either Wt or Het, indicating that the subsequent TNBS-induced reductions in cell numbers observed in* Fads1 *Null mice were not a byproduct of reduced splenic T cell numbers or activation. The reduced percentage of splenic Th17 and Th1 cells in* Fads1* Null mice coincided with reduced colonic AA-derived eicosanoid levels including PGE_2_ (Table 1) and reduced colonic injury scores (Figure 5(d)). Interestingly, both the* Fat-1* and* Fads1* Null TNBS-treated mice exhibited suppressed IL-17A, IL-23, and ROR*γ* *τ* colonic mRNA levels compared to Wt littermate controls, consistent with reduced inflammation and Th17 cell polarization, function, and maintenance (Tables 3 and 4) [57, 63, 65]. Collectively, the mucosal gene expression data indicate that the local percentage of activated Th17 cells in the colon in response to TNBS-induced colitis was likely also reduced in the* Fads1* Null mouse, similar to the outcome in the* Fat-1* mouse, although future studies confirming this interpretation are required.

AA is enriched in the membrane phospholipids of cells involved in the inflammatory response, and therefore, this fatty acid serves as the major precursor for eicosanoid mediators [66], which modulate both the intensity and duration of inflammatory responses [67]. The AA-derived eicosanoids have been shown to exert largely proinflammatory effects, although some anti-inflammatory effects have been reported [66, 67]. Ultimately, the eicosanoid profile and resulting functional outcome are contextually dependent upon the cell types present within the tissue microenvironment and the nature of the inflammatory stimulus. In this study, we showed that antagonism of AA-derived eicosanoids (specifically PGE_2_) by two distinct mechanisms results in fewer proinflammatory Th1 and Th17 cells both in local colitis-associated tissue sites and, systemically, a remodeled colonic phenotype consistent with reduced Th17 cell activation and maintenance, and reduced TNBS-induced mucosal damage and disease severity. PGE_2_-mediated reduction of mucosal damage has been reported in the dextran sodium sulfate- (DSS-) induced acute colitis model [68, 69]. This beneficial effect may be mediated through the EP4 receptor [70, 71] and is likely attributable to the ability of PGE_2_ to stimulate epithelial cell proliferation in the presence of DSS [72]. However,* Fads1* Null mice, which are devoid of PGE_2_, exhibit reduced viability in response to DSS and are unable to stimulate mucosal repair mechanisms [48]. Since DSS is toxic to the gut epithelial cells and affects the integrity of the mucosal barrier [49], it is more appropriately utilized as a mucosal wounding model, which is further supported by the lack of adaptive immune system involvement in the acute phase [73], thereby questioning the applicability of the DSS model to human IBD.

Conversely, the TNBS colitis model recapitulates many of the macroscopic and histological characteristics of human IBD [74, 75]. CD4^+^ T cell activation/tissue accumulation is a key feature of the immunopathology [49, 74, 75], and specifically, the Th17 cell subset has been shown to drive the inflammatory pathology [23]. Moreover, inhibition of CD4^+^ T cells via CD4 mimetics or anti-CD4 monoclonal antibody prevents disease development [76]. Administration of 16,16-dimethyl prostaglandin E_2_ or Enprostil (PGE_2_ analogue) to TNBS-treated rats has been shown to acutely reduce histological damage scores and myeloperoxidase activity [77, 78], although these effects are not sustained for longer durations after exposure [78]. However, Th17 cell activation and Th17 cell driven inflammation mediated by PGE_2_ during TNBS-induced colitis have been documented previously [18, 26–29]. Other AA-derived eicosanoids may play a contributing role to the activation of Th17 cells as documented elsewhere [32]. Therefore, future studies investigating the specific contributions of other n-6-derived eicosanoids are warranted. The effects of PGE_2_ are mediated through receptors (EP1-EP4), which are expressed on multiple cell types [79] including T cells, dendritic cells [25, 26, 28], and the colonic mucosa [80]. Previous data indicate that the PGE_2_-dependent effect on Th17 cells is meditated through the EP2 and EP4 receptors [25, 26, 29], suggesting that antagonism of these receptors could be a potential therapeutic target for IBD treatment, although future studies are required.

In conclusion, our observations demonstrate the utility of antagonizing colonic AA-derived eicosanoids as a mechanism to reduce inflammatory T cell activation and improve colitis-associated immunopathology. Moreover, these results suggest that dietary n-3 PUFA could be used alone, or as an adjunctive therapy, in improving the clinical outcome of colonic mucosal Th17 cell-mediated pathologies.

## Supplementary Material

Final body weights and colon lengths of saline control and TNBS-treated Fat-1 and Fads1 mice are shown in Supplemental Tables 1 and 2, respectively. Representative dot plots depicting the detection of CD4^+^ T cell subsets in TNBS-treated Fat-1 and Fads1 mice are shown in Supplemental Figures 1 and 2, respectively.

## Figures and Tables

**Table 1:** Colonic mucosal eicosanoid and cannabinoid profiles in Wt and *Fat-1* TNBS-treated mice^1^.

Eicosanoid (ng/mg protein)	Wt	*Fat-1 *
PGE_1_	0.68 ± 0.16	1.00 ± 0.35
PGE_2_	13.79 ± 3.46	7.50 ± 1.79∗
PGE_3_	0.00 ± 0.00	0.24 ± 0.03∗
PGD_2_	44.37 ± 2.09	23.18 ± 5.69∗
PGF_2*α*_	4.16 ± 0.64	2.40 ± 0.59∗
6-keto-PGF_1*α*_	3.65 ± 0.73	2.05 ± 0.35
TXB_2_	1.02 ± 0.16	0.85 ± 0.28
13-PGE_1_	0.02 ± 0.01	0.00 ± 0.00
13-PGE_2_	11.33 ± 2.36	7.68 ± 1.27
13-HODE	12.29 ± 2.68	8.33 ± 0.87
5-HETE	1.01 ± 0.18	0.93 ± 0.35
12-HETE	7.74 ± 2.22	8.40 ± 3.69
15-HETE	0.97 ± 0.13	0.50 ± 0.15∗
AEA	44.10 ± 3.89	19.97 ± 6.35∗
2-AG	0.50 ± 0.05	0.24 ± 0.03∗

^1^Mean values ± SEM of TNBS-treated *Fat-1* mice (*n* = 4-5/genotype) and values marked with an asterisk differ from Wt (*P* ≤ 0.05). PG, prostaglandin; TX, thromboxane; 13-PGE_1_, 13,14-dihydro 15-keto-PGE_1_; 13-PGE_2_, 13,14-dihydro 15-keto-PGE_2_; HODE, hydroxyl-octadecadienoic acid; HETE, hydroxyl-eicosatetraenoic acid; AEA, arachidonoyl ethanolamine; and AG, arachidonoyl glycerol.

**Table 2:** Colonic mucosal eicosanoid and cannabinoid profiles in *Fads1* TNBS-treated mice^1^.

Eicosanoid (ng/mg protein)	Wt	Het	Null
PGE_1_	1.91 ± 1.09∗	1.87 ± 0.50∗	16.36 ± 6.88^‡^
PGE_2_	15.70 ± 4.92∗	14.19 ± 1.66∗	1.92 ± 0.77^‡^
PGE_3_	0.00 ± 0.00	0.00 ± 0.00	0.00 ± 0.00
PGD_2_	43.12 ± 4.51∗	39.14 ± 2.51∗	1.38 ± 0.06^‡^
PGF_2*α*_	5.94 ± 0.98∗	4.15 ± 0.57∗	0.11 ± 0.03^‡^
6-keto-PGF_1*α*_	3.78 ± 0.98∗	3.00 ± 0.74∗	0.41 ± 0.11^‡^
TXB_2_	1.08 ± 0.23∗	1.26 ± 0.21∗	0.17 ± 0.02^‡^
13-PGE_1_	0.11 ± 0.05∗	0.07 ± 0.03∗	0.78 ± 0.14^‡^
13-PGE_2_	14.34 ± 1.62∗	7.71 ± 0.94^‡^	0.69 ± 0.09^§^
13-HODE	14.05 ± 2.63	23.18 ± 7.63	9.25 ± 1.67
5-HETE	1.04 ± 0.16∗	2.65 ± 1.35∗	0.12 ± 0.01^‡^
12-HETE	6.47 ± 1.88∗	47.66 ± 12.84^‡^	2.57 ± 1.40∗
15-HETE	1.06 ± 0.10	3.04 ± 1.50	0.11 ± 0.02
AEA	0.59 ± 0.04∗	0.52 ± 0.07∗	0.04 ± 0.00^‡^
2-AG	34.88 ± 6.74∗	56.17 ± 12.53∗	2.32 ± 0.57^‡^

^1^Mean values ± SEM of TNBS-treated Wt, Het, and Null *Fads1* mice (*n* = 5/genotype). Values not sharing a superscript symbol differ (*P* ≤ 0.05). PG, prostaglandin; TX, thromboxane; 13-PGE_1_, 13,14-dihydro 15-keto-PGE_1_; 13-PGE_2_, 13,14-dihydro 15-keto-PGE_2_; HODE, hydroxyl-octadecadienoic acid; HETE, hydroxyl-eicosatetraenoic acid; AEA, arachidonoyl ethanolamine; and AG, arachidonoyl glycerol.

**Table 3:** Colonic mucosal mRNA expression in Wt and *Fat-1* TNBS-treated mice^1^.

Gene	Wt	*Fat-1 *
IL-17A	6.54 ± 2.95	2.04 ± 0.73∗
IL-17F	6.14 ± 1.33	5.96 ± 1.03
IL-21	0.63 ± 0.09	0.15 ± 0.07∗
IL-22	0.48 ± 0.17	0.95 ± 0.32
IL-23	1.63 ± 0.42	1.01 ± 0.41
IL-23R	1.72 ± 0.44	1.76 ± 0.28
IL-27	3.68 ± 0.84	7.50 ± 1.49∗
CCL20	4.93 ± 1.59	4.25 ± 0.61
CCR6	0.45 ± 0.97	0.7 ± 0.48
ROR*γτ*	15.79 ± 1.29	12.25 ± 0.82∗
Tbet	2.30 ± 0.79	1.42 ± 0.46
Foxp3	1.74 ± 0.97	3.24 ± 0.63
IL-10	1.08 ± 0.68	2.64 ± 0.52∗
TGF*β*1	5.56 ± 0.66	6.91 ± 2.26
MCP-1	8.34 ± 3.75	3.03 ± 0.58∗
IL-1*β*	1.28 ± 0.87	0.39 ± 0.09∗
TNF*α*	1.90 ± 1.3	0.73 ± 0.18∗
IL-6	2.88 ± 0.86	2.66 ± 0.61
IFN*γ*	0.63 ± 0.22	0.34 ± 0.15
IL-4	0.20 ± 0.11	0.28 ± 0.25

^1^Mean values ± SEM of TNBS-treated Wt and *Fat-1* mice (*n* = 8–10/genotype). All genes were measured within the same samples and expression was normalized to ribosomal 18S (in arbitrary units). For each gene, values marked with an asterisk differ from Wt (*P* ≤ 0.05).

**Table 4:** Colonic mucosal mRNA expression in Wt, Het, and Null *Fads1* TNBS-treated mice^1^.

Gene	Wt	Het	Null
IL-17A	7.62 ± 4.70∗	1.10 ± 0.32^‡^	0.31 ± 0.01^‡^
IL-17F	12.95 ± 3.71∗	5.46 ± 1.07^‡^	4.19 ± 0.98^‡^
IL-21	1.53 ± 0.80	0.70 ± 0.18	0.47 ± 0.14
IL-22	1.48 ± 1.05	0.44 ± 0.33	0.10 ± 0.10
IL-23	3.88 ± 0.43∗	3.58 ± 0.57^∗‡^	2.19 ± 0.39^‡^
IL-23R	0.73 ± 0.16	0.40 ± 0.05	0.65 ± 0.21
IL-27	2.75 ± 0.67	1.86 ± 0.42	1.80 ± 0.46
CCL20	1.42 ± 0.49	1.36 ± 0.57	1.13 ± 0.30
CCR6	1.22 ± 0.38	0.82 ± 0.24	0.91 ± 0.44
ROR*γτ*	11.07 ± 2.04∗	8.75 ± 1.34∗	5.28 ± 0.17^‡^
Tbet	8.35 ± 1.32∗	3.30 ± 0.66^‡^	4.12 ± 0.69^‡^
Foxp3	2.95 ± 0.65	2.52 ± 0.23	1.81 ± 0.27
IL-10	13.57 ± 3.91∗	9.08 ± 2.77∗	3.95 ± 1.38^‡^
TGF*β*1	1.80 ± 0.27	1.68 ± 0.26	1.31 ± 0.13
MCP-1	25.57 ± 8.37∗	32.41 ± 14.00∗	8.86 ± 3.49^‡^
IL-1*β*	44.13 ± 12.57	31.30 ± 13.19	24.51 ± 12.95
TNF*α*	9.85 ± 1.81	9.89 ± 3.82	5.65 ± 1.72
IL-6	11.54 ± 4.07∗	7.11 ± 3.34^∗‡^	2.10 ± 0.73^‡^
IFN*γ*	1.89 ± 1.01	0.52 ± 0.16	0.62 ± 0.21
IL-4	0.64 ± 0.36	nd	0.38 ± 0.73

^1^Mean values ± SEM of TNBS-treated Wt, Het, and Null *Fads1* mice (*n* = 6–9/genotype). All genes were measured within the same samples and expression was normalized to ribosomal 18S (in arbitrary units). For each gene, values not sharing a superscript symbol differ between genotypes (*P* ≤ 0.05). nd, not detected.

## Abbreviations

IBD: Inflammatory bowel disease
CD: Crohn's disease
FO: Fish oil
PUFA: Polyunsaturated fatty acids
EPA: Eicosapentaenoic acid
DHA: Docosahexaenoic acid
Tregs: Regulatory T cells
TNBS: 2,4,6-Trinitrobenzene sulfonic acid
PGE_2_: Prostaglandin E_2_
PG: Prostaglandin
TX: Thromboxane
HODE: Hydroxyl-octadecadienoic acid
HETE: Hydroxyl-eicosatetraenoic acid
AEA: Arachidonoyl ethanolamine
AG: Arachidonoyl glycerol
DSS: Dextran sodium sulfate.
