# Supplementary material for: Antagonizing Arachidonic Acid-Derived Eicosanoids Reduces Inflammatory Th17 and Th1 Cell-Mediated Inflammation and Colitis Severity
Source: Mediators Inflamm. 2014 Jul 17;2014:917149. doi: 10.1155/2014/917149 (PMC4127240; doi:10.1155/2014/917149)
Supplement: Supplementary file 1 — Final body weights and colon lengths of saline control and TNBS-treated Fat-1 and Fads1 mice are shown in Supplemental Tables 1 and 2, respectively. Representative dot plots depicting the detection of CD4+ T cell subsets in TNBS-treated Fat-1 and Fads1 mice are shown in Supplemental Figures 1 and 2, respectively. [file 917149.f1.pdf]

Supplemental Table 1. Final body weights and colon lengths of Wt and *Fat-1* saline control and TNBS-treated mice[1]

	Saline Control		TNBS	
	**Wt**	***Fat-1***	**Wt**	***Fat-1***
Body Weight (g)	24.09 ± 0.66*	23.01 ± 0.82*	18.75 ± 0.64‡	19.89 ± 0.84‡
Colon Length (cm)	6.11 ± 0.16*	5.92 ± 0.20*	4.82 ± 0.13‡	5.14 ± 0.52‡

[1]Mean values ± SEM of saline control (n=4/genotype) and TNBS-treated Wt and *Fat-1* mice (n=9/genotype). Within rows, values not sharing a superscript symbol differ (*P*≤0.05).

Supplemental Table 2. Final body weights and colon lengths *Fads1* saline control and TNBS-treated mice[1]

	Saline Control			TNBS		
	**Wt**	**Het**	**Null**	**Wt**	**Het**	**Null**
Body Weight (g)	22.53±1.66*	22.13±1.20*	19.23±1.18*	18.86±1.23‡	16.39±0.86‡	16.75±1.14‡
Colon Length (cm)	6.40±0.13*	6.10±0.12*	6.30±0.12*	5.53±0.26‡	5.33±0.25‡	5.48±0.32‡

[1]Mean values ± SEM of Wt, Het and Null *Fads1* saline control (n=4/genotype) and TNBS-treated mice (n=6-9/genotype). Values marked with a different superscript symbol differ ($P \leq 0.05$).

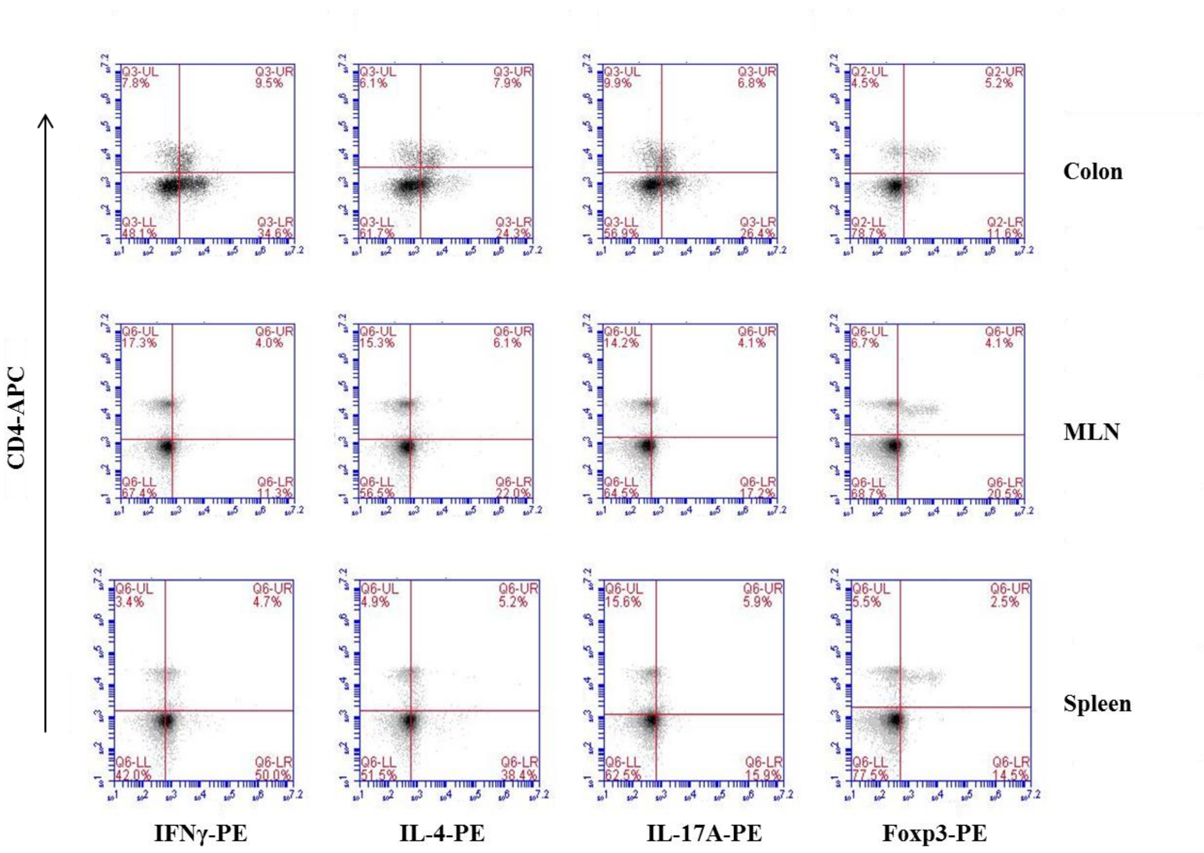

Supplemental Figure 1. Representative dot plots deplicting the detection of $CD4^+$ T cell subsets in TNBS-treated Wt colon lamina propria (top row), MLN (middle row) and spleen (bottom row). All cells were surface stained with CD4-APC (y axis) followed by intracelluar detection of T cell subset specfic markers (x axis): IFNγ-PE (Th1), IL-4-PE (Th2), IL-17A-PE (Th17) and Foxp3-PE (Treg) (see Materials and Methods). Flow cytometric analyses were conducted using an Accuri C6 flow cytometer (Accuri Cytometers).

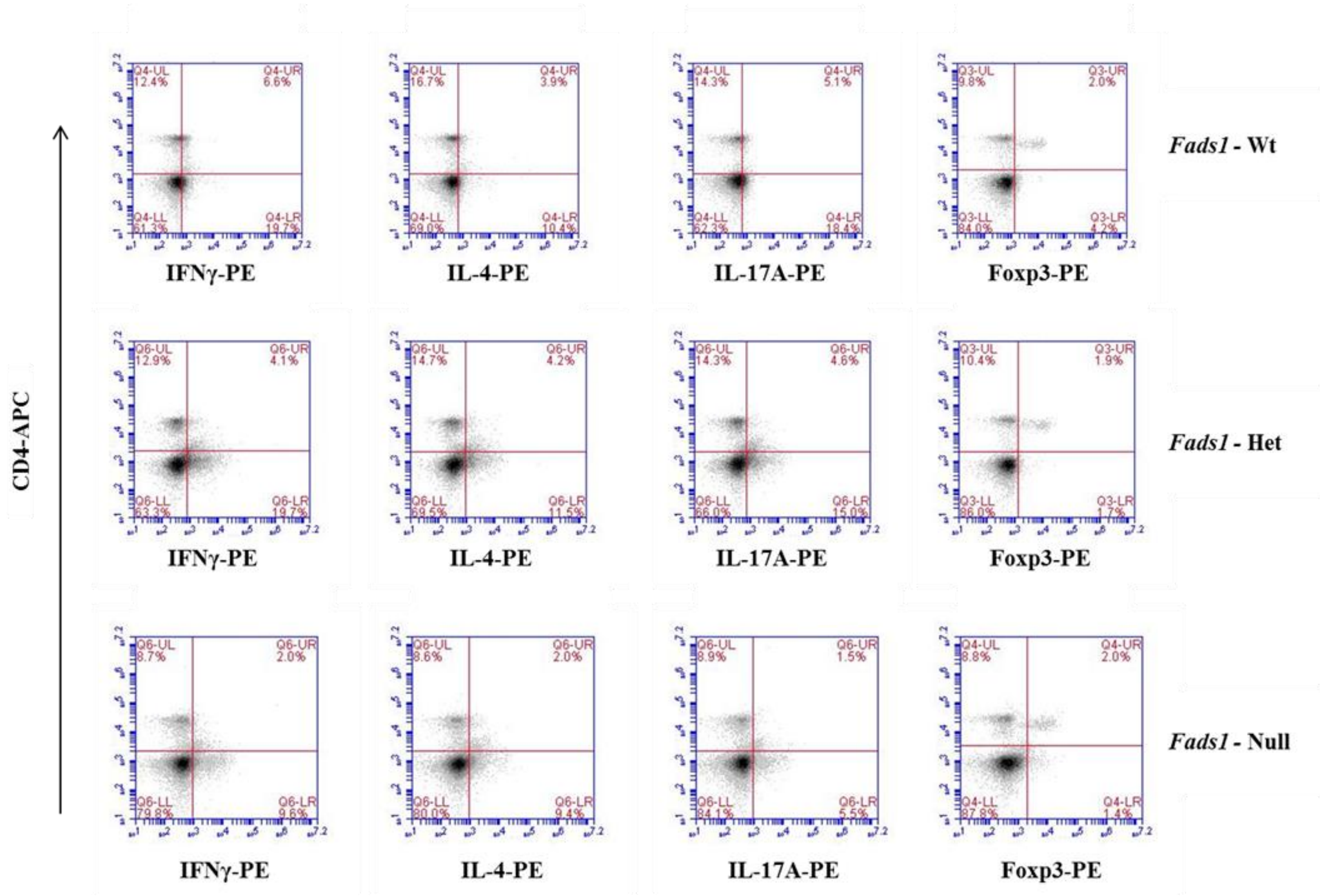

Supplemental Figure 2. Representative dot plots deplicting the detection of splenic $CD4^+$ T cell subsets in TNBS-treated *Fads1* Wt (top row), Het (middle row) and Null mice (bottom row). All cells were surface stained with CD4-APC (y axis) followed by intracelluar detection of T cell subset specfic markers (x axis): IFNγ-PE (Th1), IL-4-PE (Th2), IL-17A-PE (Th17) and Foxp3-PE (Treg) (see Materials and Methods). Flow cytometric analyses were conducted using an Accuri C6 flow cytometer (Accuri Cytometers).
